# Triptolide Inhibits Th17 Response by Upregulating microRNA-204-5p and Suppressing STAT3 Phosphorylation in Psoriasis

**DOI:** 10.1155/2022/7468396

**Published:** 2022-11-17

**Authors:** Qi He, Xingyue Wu, Quan Shi

**Affiliations:** ^1^Department of Dermatology, Hubei Provincial Hospital of Traditional Chinese Medicine, The Affiliated Hospital of Hubei University of Chinese Medicine, Wuhan 430061, Hubei, China; ^2^Department of Dermatology, Hubei Province Academy of Traditional Chinese Medicine, Wuhan 430074, Hubei, China

## Abstract

**Background:**

Psoriasis is an immune and inflammation-related skin disease. Triptolide with immunosuppressive and anti-inflammatory properties has been utilized for psoriasis treatment. However, the potential immunological mechanisms of triptolide have not been fully elucidated.

**Methods:**

Using an imiquimod (IMQ)-induced psoriatic mouse model, we detected the effects of triptolide on psoriasis-like lesions including scales, thickening, and erythema. Methyl thiazol tetrazolium (MTT) cytotoxicity assay was performed for evaluating the influence of triptolide on cell viability. Gene expression at mRNA and protein levels were examined by reverse transcription-quantitative polymerase chain reaction and Western blot analysis, respectively. The combination between microRNA-204-5p (miR-204-5p) and signal transduction and transcription activator-3 (STAT3) was confirmed by luciferase reporter assay. Enzyme-linked immunosorbent assay was conducted to examine interleukin (IL)-17 and interferon-*γ* (IFN-*γ*) levels using corresponding kits. Hematoxylin and eosin staining was used for the visualization of epidermal thickness. Flow cytometry analysis was employed for examining *T* helper (Th) 17 cells.

**Results:**

Triptolide ameliorated IMQ-induced psoriatic skin lesions manifested by the decreased psoriasis area and severity indexes (PASI) scores. Triptolide inhibited Th17 cell differentiation from splenocytes. Additionally, triptolide elevated miR-204-5p expression, whereas it downregulated STAT3 expression levels both *in vitro* and *in vivo*. Moreover, miR-204-5p directly targeted STAT3 in HaCaT cells. Furthermore, triptolide repressed the expression of proinflammatory cytokines in IMQ-evoked psoriasis-like mice.

**Conclusion:**

Triptolide inhibits STAT3 phosphorylation via upregulating miR-204-5p and thus suppressing Th17 response in psoriasis.

## 1. Introduction

Psoriasis is one of the chronic and immune-mediated disease, characterized by hyperplasia of the epidermis, dermal angiogenesis, and infiltration of inflammatory cells, affecting approximately 2-3% population worldwide [1, 2]. Psoriasis can develop into psoriatic arthritis, leading to a variety of complications such as cancer, cardiovascular disease, obesity, Crohn's disease, and psoriatic arthritis [3, 4]. Various factors are associated with psoriasis occurrences such as immunity, metabolic disorders, and environmental and genetic factors [5]. To date, the pathogenesis of psoriasis has not been fully elucidated, but it is widely accepted that *T* cell-mediated immunity exerts a crucial function in the development of psoriasis. Additionally, the imbalance between anti-inflammatory Treg cells and proinflammatory *T* helper (Th) 17 cells is responsible for multiple autoimmune diseases, including psoriasis [6]. Th17 cells can produce interleukin-17 (IL-17) that has been illuminated to elicit a pivotal function in psoriasis inducing the expression of antimicrobial peptides, cytokines, and proinflammatory chemokines in keratinocytes [7]. Although conventional immunosuppressants and/or glucocorticosteroids for psoriatic patients bring about temporary alleviation, the side effects for these patients are quite serious [8, 9]. Therefore, it is urgently needed to identify the etiology of psoriasis and recognize novel and effective drugs and/or targets for the treatment of psoriasis patients.

MicroRNAs (miRNAs) are small noncoding RNA molecules containing about 22 nucleotides. The critical function of miRNAs in the modulation of RNA silencing and gene expression at the posttranscriptional level has been proved [10, 11]. In addition, miRNAs are involved in various biological processes, and their aberrant expression could lead to many human diseases such as immune abnormalities, inflammations, and carcinomas [12, 13]. Numerous miRNAs such as miR-138 [14], miR-31 [15], and miR-203 [16] have participated in psoriasis development and are dysregulated in keratinocytes and lymphocytes from patients with psoriasis. miR-204-5p is shown to be related to immune and inflammation [17, 18]. However, the role of miR-204-5p in psoriasis remains unclear.

Triptolide is a compound extracted from a Chinese medicinal herb *Tripterygium wilfordii* hook F (TWHF), possessing potential properties such as antitumor, immunosuppression, and anti-inflammation [19]. TWHF has been proven to have immunoregulation effects and is considered a promising drug in the treatment of systemic lupus erythematosus and rheumatoid arthritis [20–23]. In recent years, the role of triptolide in psoriasis has been uncovered. Studies have demonstrated that triptolide significantly represses the proliferation of HaCaT Cells *in vitro* and attenuates psoriatic skin inflammation induced by imiquimod (IMQ) *in vivo* [24, 25]. Importantly, clinical observation of the triptolide effect on psoriasis vulgaris reveals that the total effective rate of triptolide is up to 75.7% among the 103 patients within the treatment period [26]. A previous literature study validates that 100 nM triptolide upregulates miR-204 expression in S2-VP10 cells [27]. However, the exact dosage of triptolide applied to psoriasis patients and the molecular mechanism of its possible effects remain unclear.

In the current study, we aimed to study the biological function of triptolide and its related mechanism by which it regulates psoriasis development. We made a hypothesis that triptolide may alleviate psoriatic symptoms of mouse models and inhibit inflammatory response via regulating miR-204-5p.

### 1.1. Aims

In the current study, we aimed to study the biological function of triptolide and its related mechanism by which it regulates psoriasis development.

## 2. Materials and Methods

### 2.1. Animals

Vital River Co. Ltd. (Beijing, China) provided the BALB/*c* male mice (6–10 weeks old, 18–20 g), which were kept under specific pathogen-free conditions with food and water *ad libitum*. The Animal Ethics Committee of Hubei Provincial Hospital of Traditional Chinese Medicine and the Affiliated Hospital of Hubei University of Chinese Medicine (Hubei, China) approved all animal experiments. This study was conducted in line with the national guidelines for the care and use of laboratory animals.

### 2.2. Establishment of Psoriatic Mouse Models

The dorsal area (2 × 3 cm^2^) of mice was shaved. A total of 40 mice were randomly divided into four groups (10 per group) including the sham group, model group, model + triptolide (*L*) group, and model + triptolide (H) group. Normal control mice (sham) were applied with appropriate vaseline on the exposed back each day and intragastric administrated with saline (0.4 ml/day) for 8 continuous days. The mice in the model group were applied 42 mg of 5% imiquimod (IMQ) cream (GG0080, BIOSIC, Ding Zhou Biosic Biotechnology Co., Ltd., China) on the shaved dorsal area daily and intragastric administrated with saline (0.4 ml/day) for 8 continuous days. The mice in the model + triptolide (*L*) and model + triptolide (H) groups were applied 42 mg of 5% IMQ on the exposed back skin daily and treated with low and high doses of triptolide (10 and 40 mg/kg, respectively) and received intragastric administration of triptolide (0.4 ml/day) for 8 continuous days.

### 2.3. Psoriasis Area and Severity Indexes (PASI) Scoring

PASI scoring was used to evaluate the severity of skin inflammation and lesion by measuring erythema, scaling, and thickness. PASI scores of each category were scored independently on a scale from 0 to 4: “0” indicates none, “1” indicates “slight,” “2” indicates “moderate,” “3” indicates “marked,” and “4” indicates “severe.” The severity of the lesion was reflected via the cumulative scores of erythema, scaling, and thickening. PASI scores were scored from the day when IMQ was first administered to the last administration during the 8 consecutive days.

### 2.4. Methyl-Thiazol Tetrazolium (MTT) Cytotoxicity Assay

Following isolating splenocytes from mouse models, increased concentrations of triptolide (0–500 nM) was used to cultivate with the isolated splenocytes for 3 d. After exposure to continuous drugs, MTT (M2003, Sigma Aldrich, St Louis, MI, USA) was added into cultures. Dissolving insoluble formazan complex in DMSO was performed prior to the measurement of the absorbance at 490 nm.

### 2.5. Cell Treatment and Transfection

Human keratinocytes (HaCaT) were commercially procured from Shanghai EK-Bioscience Biotechnology Co., Ltd. HaCaT cells and the isolated splenocytes from mice were maintained in Dulbecco's modified eagle's medium (DMEM, Sigma Aldrich) containing 10% fetal bovine serum (FBS; F2442, Sigma Aldrich). A humidified incubator with 5% CO_2_ at 37°C was used to incubate the cells. The isolated splenocytes were resuspended in DMEM (Sigma Aldrich) supplemented with 10% FBS (Sigma Aldrich). Splenocytes were stimulated with anti-CD3 (1 lg/ml; BD pharMingen, San Diego, CA, USA) or anti-CD3 plus interleukin (IL)-6 (20 ng/ml; peproTech, London, UK) and transforming growth factor-*β* (TGF-*β*; 1 ng/ml; PeproTech) in the presence or absence of triptolide for 4 d.

### 2.6. Cell Transfection

MiR-204-5p mimics were designed and synthesized to upregulate the expression of miR-204-5p with NC mimics as the corresponding control. Transfection of plasmids was performed for 48 h using Lipofectamine 2000 (Invitrogen, USA) in accordance with the supplier's guidelines. The synthetical plasmids including miR-204-5p mimics and NC mimics were procured from Genepharma in Shanghai, China. Increased concentrations of triptolide (1-100 nM) were used to treat the cells for 24 h. Triptolide was prepared as previously delineated and stored at −20°C [28].

### 2.7. Western Blot Analysis

After lysing the cells using RIPA lysis buffer (P0013C, Beyotime Biotechnology, Shanghai, China) on ice for 10 min, a BCA protein assay kit (P0010, Beyotime Biotechnology) was employed for determining protein concentrations. Then, the protein samples were subjected to 10% sodium dodecyl sulfate-polyacrylamide gel electrophoresis (SY4584, YITABIO, Beijing, China) and transferred onto polyvinylidene fluoride membranes (S25907, Shanghai Yuanye Bio-Technology Co., Ltd., Shanghai, China) in a conventional way. Afterwards, nonfat milk powder was used to block the membrane, which was incubated with primary antibodies against signal transducer and activator of transcription 3 (STAT3; 9139, Cell Signaling Technology), p-STAT3 (Tyr705) (9145, Cell Signaling Technology), and glyceraldehyde 3-phosphate dehydrogenase (GAPDH; ab181603, Abcam) in a 4°C refrigerator overnight. The next day, the membrane was incubated with a secondary antibody (ab288151, Abcam) at room temperature for 1 h. GAPDH functioned as the loading control. Clarity™ Western ECL substrate (1705061, Bio-Rad laboratories, Shanghai, China) was employed for visualization of the protein bands. Quantity One software was utilized to quantify protein band density.

### 2.8. Reverse Transcription-Quantitative Polymerase Chain Reaction (RT-qPCR)

TRIzol reagent (Takara, Dalian, China) was used for extracting total RNA. Reverse transcription of RNAs into complementary DNA (cDNA) was achieved with the employment of an SYBR Green Master Mix Kit (Takara). PCR was performed with the SYBR Prime Script RTPCR Kit (Takara) on a CFX96™ real-time system (Bio-Rad). GAPDH and U6 were used as internal references for miRNAs and mRNAs, respectively. The 2^−ΔΔCt^ method was utilized for calculating relative gene levels. The primers were as follows: miR-204-5p f: 5ʹ-UUCCCUUUGUCAUCCUAUGCCU-3ʹ, r: 5′-CTCAACTGGTGTCGTGGA-3ʹ; STAT3, f: 5ʹ-AAAGTATTGTCGGCCAGAG-3ʹ, r: 5ʹ- CATCAATGAATGGTGTCACAC-3ʹ; IL-6 f: 5ʹ-ACTTCCATCCAGTTGCCTTCTTGG-3ʹ, r: 5ʹ-TTAAGCCTCCGACTTGTGAAGTGG-3ʹ; IL-17A f: 5ʹ-CAGACTACCTCAACCGTTCCA-3ʹ, r: 5ʹ-ACAATCGAGGCCACGCAGGTGCAGC-3ʹ; IL-17F f: 5ʹ-TGCTACTGTTGATGTTGGGAC-3ʹ, r: 5ʹ-AATGCCCTGGTTTTGGTTGAA-3ʹ; TNF-*α* f: 5ʹ-AAATGGGCTCCCTCTCATCAGTTC-3ʹ, r: 5ʹ-TCTGCTTGGTGGTTTGCTACGAC-3ʹ; GAPDH f: 5ʹ-CATCAAGAAGG TGGTGAAGCAG-3ʹ, r: 5ʹ-CGTCAAAGGTGGAGGAGTGG-3ʹ; and U6, f: 5ʹ-GCGCGTCGTGAAGCG TTC-3ʹ, r: 5ʹ-GTGCAGGGTCCGAGG-3ʹ.

### 2.9. Enzyme-Linked Immunosorbent Assay (ELISA)

Splenocytes isolated from BALB/*c* mice were stimulated with anti-CD3 or anti-CD3 + IL-6 + TGF-*β* with or without 1.25–10 nM triptolide treatment for 4 days (primary culture). The supernatants were assayed for IL-17 using a mouse IL-17 ELISA kit (ab100702, Abcam).

Serum concentrations of IL-17 and interferon (IFN)-*γ* were detected using the mouse IL-17 ELISA kit (ab100702, Abcam) and a mouse IFN-*γ* ELISA kit (ab252363, Abcam), respectively.

### 2.10. Flow Cytometry Analysis

In accordance with the manufacturer's instructions, CD4^+^ T cells were isolated from splenocytes by magnetic cell sorting with mouse anti-CD4 (L3T4) microbeads (Miltenyi Biotec, Bergisch Gladbach, Germany) on an MS column. The fluorescence-activated cell sorter (FACS) was used for the determination of the purity (>96%) of CD4^+^ T cells. Immobilized anti-CD3 (1 lg/ml) together with IL-6 (20 ng/ml) and TGF-*β* (1 ng/ml) were used for stimulating CD4^+^ T cells (3 × 10^5^ cells/ml) in the presence or absence of triptolide treatment for 96 h.

The cells collected from the spleen and draining lymph node (LN) of indicated mouse models were cultured for 72 h, followed by stimulation and induction for secreting intracellular cytokines by brefeldin A (BFA; 10 *µ*g/ml), ionomycin (1 *µ*g/ml), and PMA (10 ng/ml). Following culturation for 6 h, the cells were subjected to incubation with an anti-mouse CD4-FITC antibody. Then, the cells were stained with APC-labeled anti-mouse IL-17 antibody after permeabilization. Later, a flow cytometer was adopted for analyzing CD4^+^ IL-17^+^ T cell percentage.

### 2.11. Luciferase Reporter Assay

The wild-type STAT3 reporter (STAT3-WT) and the mutant STAT3 reporter (STAT3-Mut) were generated by subcloning the 3′-UTR sequences of STAT3 bracketing the predicted miR-204-5p binding site and the full-length sequences of STAT3-Mut into the pmirGLO vectors (WN-13185, Wuhan Warner Biotechnology Co., Ltd., Hubei, China). STAT3-WT or STAT3-Mut were transfected with NC mimics or miR-204-5p mimics into HaCaT cells for 48 h. Later, the luciferase activities were examined with the employment of a dual-luciferase reporter assay system (Promega, USA).

### 2.12. Hematoxylin and Eosin (H&E) Staining

On day 8, back skin samples were collected for visualizing epidermal thickness. First, 4% neutral paraformaldehyde-fixed skin samples were embedded in paraffin, followed by slicing them into 5 *μ*m-thick sections. These skin sections were stained by H&E, and the stained slides were observed under a microscope.

### 2.13. Statistical Analysis

All quantitative data obtained from at least three independent trials are shown as the means ± standard deviation and analyzed with SPSS 15.0 software. The data between the two groups were analyzed by the unpaired *t*-test with an independent sample, while the data among multiple groups were analyzed by ANOVA followed by the post hoc Bonferroni test. The threshold for statistical significance was the *p* value less than 0.05.

## 3. Results

### 3.1. Triptolide Inhibits Th17 Differentiation

**Figure 1:**
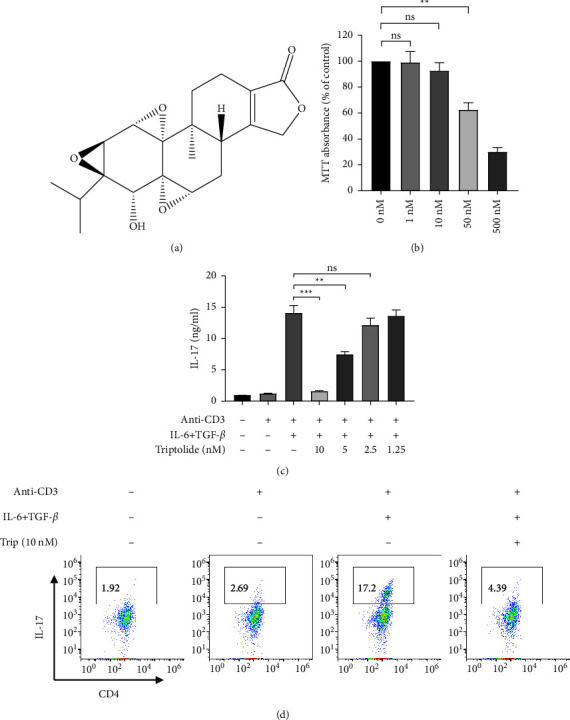
The effects of triptolide on cell viability and IL-17 differentiation. (a) Triptolide chemical structure. (b) MTT assay conducted to detect the viability of splenocytes which were isolated from mice in the presence or absence of increasing dosages of triptolide (1–500 nM) treatment. (c) Anti-CD3 or anti-CD3 + IL-6 + TGF-*β* used to stimulate the isolated splenocytes from mouse models with or without 1.25–10 nM triptolide treatment. ELISA was used for measuring IL-17 concentration in the supernatants. (d) IL-17^+^CD4^+^ T population determined by flow cytometry analysis. Each bar presents the mean ± standard deviation obtained from three to four separate experiments. ^*∗∗*^*P* < 0.01, ^*∗∗∗*^*P* < 0.001 compared to the model group. n.s indicates no significance.

MTT was first utilized to detect the influence of triptolide (Figure 1(a)) on cell viability. As a result, 1–10 nM triptolide showed no notable effect on cell viability compared with the cells treated with 0 nM triptolide, while 50 nM triptolide significantly suppressed cell viability, suggesting that the no-toxic effect of triptolide on cells was 1–50 nM (Figure 1(b)). Then, the influence of triptolide on Th17 differentiation was investigated. ELISA was performed to detect IL-17 concentration by assessing the culture supernatants. Results showed that the cultures supplemented with IL-6 and TGF-*β* led to the significant upregulation of IL-17 production. The induced production of IL-17 was then dose-dependently repressed by triptolide treatment. Triptolide at the dosage of 10 nM almost completely suppressed the production of IL-17 (Figure 1(c)). With the aim of characterizing cell subsets of IL-17-producing cells at the signal-cell level, flow cytometry analysis was employed. Compared with the anti-CD3 group, a large fraction of IL-17^+^CD4^+^ T cells was induced by anti-CD3 together with IL-6 and TGF-*β* after primary culture. However, compared with the cells without triptolide treatment, a significant decrease of IL-17^+^CD4^+^*T* population in triptolide-induced cells of primary culture was observed (Figure 1(d)). All these data demonstrated that triptolide suppresses the differentiation of Th17 cells from splenocytes.

### 3.2. Effects of Triptolide on miR-204-5p and STAT3

**Figure 2:**
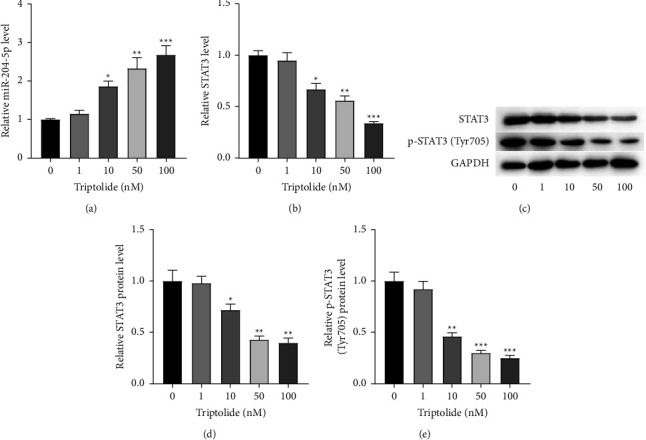
MiR-204-5p and STAT3 expression in triptolide-treated HaCaT cells *in vitro*. HaCaT cells were treated with 0–100 nM triptolide.(a and b) MiR-204-5p and STAT3 expressions in triptolide-treated HaCaT cells detected by RT-qPCR analysis. (c and e) Protein levels of STAT3 and p-STAT3 (Tyr705) tested by Western blot analysis. Each bar presents the mean ± standard deviation obtained from three to four separate experiments. Statistical significance was determined using two-way ANOVA followed by the post hoc Bonferroni test. ^*∗*^*P* < 0.05, ^*∗∗*^*P* < 0.01, ^*∗∗∗*^*P* < 0.001 compared to the model group.

The regulatory effect of triptolide on miR-204 expression has been reported. Several literature studies have also validated the modulatory effect of miR-204-5p on STAT3 expression. Additionally, STAT3 is extensively involved in Th17 cell differentiation. Thus, we intended to investigate whether miR-204-5p and STAT3 are engaged in triptolide-mediated psoriasis development. First, miR-204-5p expression was detected in HaCaT cells treated with 0–100 nM triptolide. Results of RT-qPCR delineated that triptolide upregulated miR-204-5p expression *in vitro* in a dose-dependent manner (Figure 2(a)). In contrast, STAT3 mRNA expression displayed a downward trend in triptolide-treated HaCaT cells (Figure 2(b)). Meanwhile, we observed the reduced protein levels of STAT3 and p-STAT3 caused by increased concentrations of triptolide (Figures 2(c)–2(e)). Therefore, we concluded that triptolide upregulates miR-204-5p expression, whereas it downregulates STAT3 expression levels in HaCaT cells.

### 3.3. MiR-204-5p Targets STAT3

**Figure 3:**
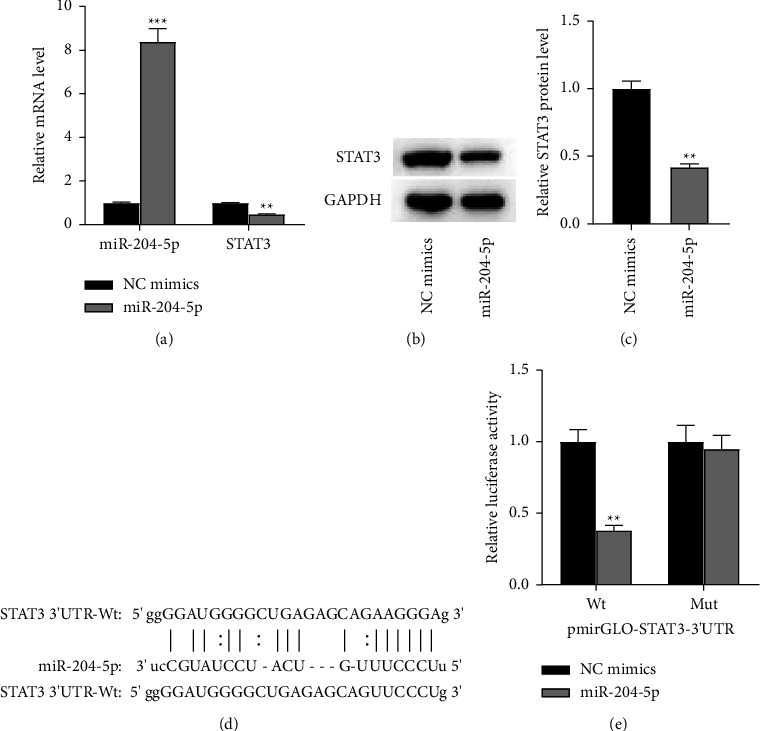
MiR-204-5p targets STAT3 in HaCaT cells. (a) Relative expression of miR-204-5p and STAT3 in HaCaT cells transfected with NC mimics or miR-204-5p mimics examined by RT-qPCR. (b and c) The STAT3 protein level in HaCaT cells with the transfection of NC mimics or miR-204-5p mimics examined by Western blot analysis. (d) Binding sequences between miR-204-5p and STAT3. (e) The combination between miR-204-5p and STAT3 validated by luciferase reporter assay. Each bar presents the mean ± standard deviation obtained from three to four separate experiments. ^*∗∗*^*P* < 0.01, ^*∗∗∗*^*P* < 0.001 compared to the model group.

To probe into whether miR-204-5p directly targets the 3′UTR of STAT3, miR-204-5p mimics were first transfected into HaCaT cells. It was found that overexpressed miR-204-5p led to an increase in miR-204-5p expression and a decrease in STAT3 expression (Figure 3(a)). Additionally, overexpression of miR-204-5p significantly inhibited STAT3 protein expression (Figures 3(b)–3(c)). To evaluate the combinative possibility of miR-204-5p with STAT3, the 3′UTR of STAT3 was cloned into a vector harboring the luciferase reporter gene. Meanwhile, six seed nucleotides AAGCGA in the mutant vector were replaced with UUCCCU to eliminate the possible recognition (Figure 3(d)). The results of the luciferase reporter assay manifested that the luciferase activity of STAT3 3′UTR-Wt was reduced to 38% upon miR-204-5p overexpression. However, overexpressed miR-204-5p caused almost no changes in the luciferase activity of STAT3 3′UTR-Mut (Figure 3(e)). Combining the results of these experiments, we demonstrated that miR-204-5p directly targets STAT3 in HaCaT cells.

### 3.4. Triptolide Alleviates IMQ-Evoked Psoriatic Skin Lesion

**Figure 4:**
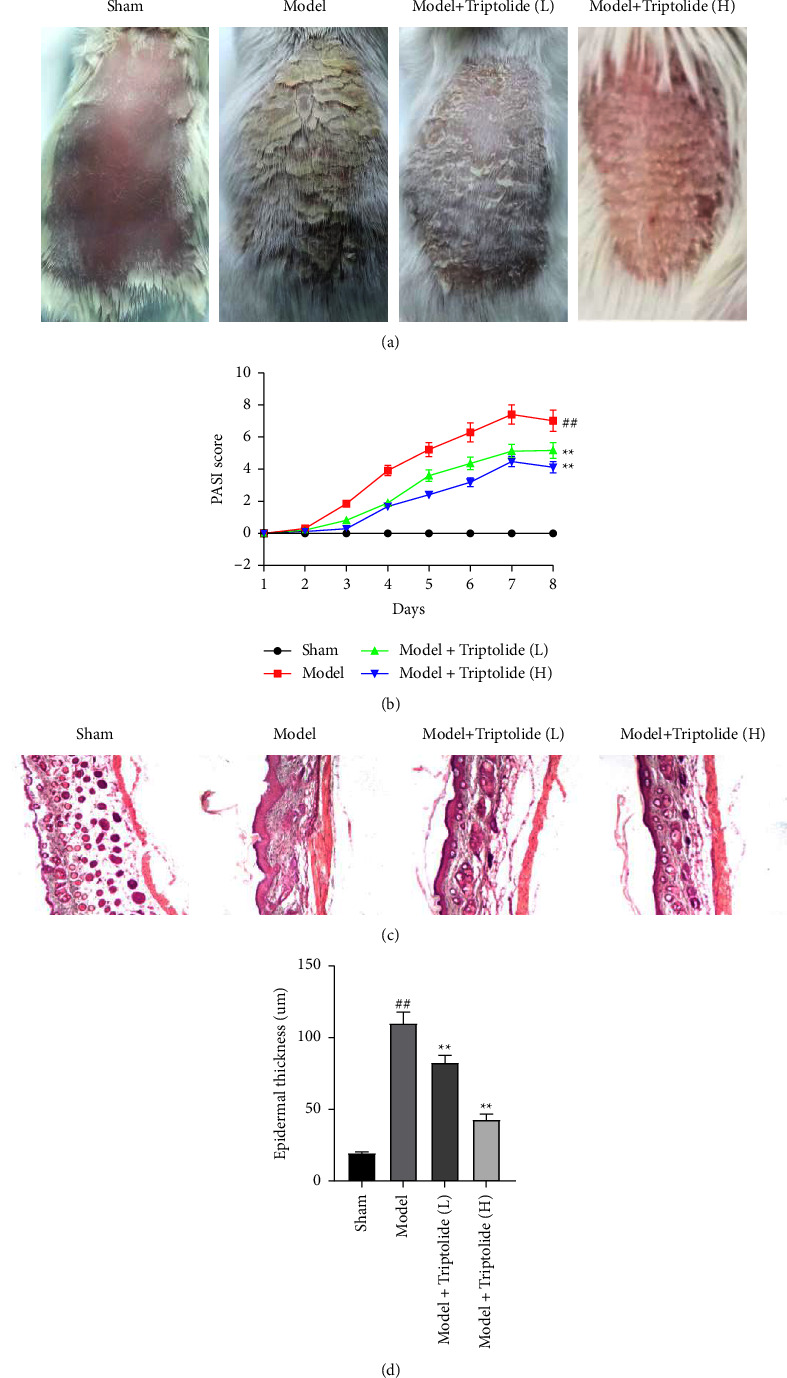
Triptolide mitigates psoriatic symptoms of psoriatic mice induced by IMQ. BALB/*c* mice in the sham group were painted with vaseline daily. Mice in the model group and triptolide-treated groups were painted with IMQ daily. The triptolide groups were applied simultaneously with IMQ and triptolide (10 or 40 mg/kg). Skin lesion analysis was performed following sacrificing these mice. (a) Back skin photos of mouse models in the sham group, model group, model + triptolide (L) group, and model + triptolide (H) group taken on the 8th day after IMQ painting. (b) PASI scores of mouse models in each group evaluated daily. (c) The H&E-stained dorsal skin of mouse models in the indicated groups after IMQ treatment for 8 days. (d) Evaluation of epidermal thickness under a microscope via ImagePro Plus software. Each column is representative of data obtained from a minimum of 8 animals. ^##^*P* < 0.01 compared to the sham group; ^*∗∗*^*P* < 0.01, ^*∗∗∗*^*P* < 0.001 compared to the model group.

A mouse model of IMQ-induced psoriasis was constructed to detect the effects of triptolide on psoriasis. Compared with the control mice (no apparent signs of skin inflammation) in the sham group, psoriatic mice treated with IMQ showed severe psoriasis-like lesions, including scales, thickening, and erythema in the model group. Severe lesions of scales, thickening, and erythema were attenuated following treatment with low doses of triptolide (10 mg/kg) and further alleviated by high doses of triptolide (40 mg/kg) (Figure 4(a)). Compared with the mice in the sham group, PASI scores were much higher in IMQ-treated psoriatic mice. However, on the 8^th^ day after treatment with low and high doses of triptolide, PASI scores were gradually reduced (Figure 4(b)). Furthermore, skin tissues from mouse models were subjected to H&E staining, which demonstrated that IMQ-induced higher epidermal thickness in the model group (109.82 ± 8.55 *μ*m) was significantly reduced by a low dose of triptolide (82.47 ± 5.63 *μ*m) and further downregulated by a high dose of triptolide (42.88 ± 4.24 *μ*m) (Figures 4(c) and 4(d)). These results suggested that psoriatic skin lesions induced by IMQ can be ameliorated by triptolide treatment.

### 3.5. Triptolide Decreases IL-17-Expressing Cell Frequency within CD4+ Cell Population in Psoriatic Mouse Models Induced by IMQ

**Figure 5:**
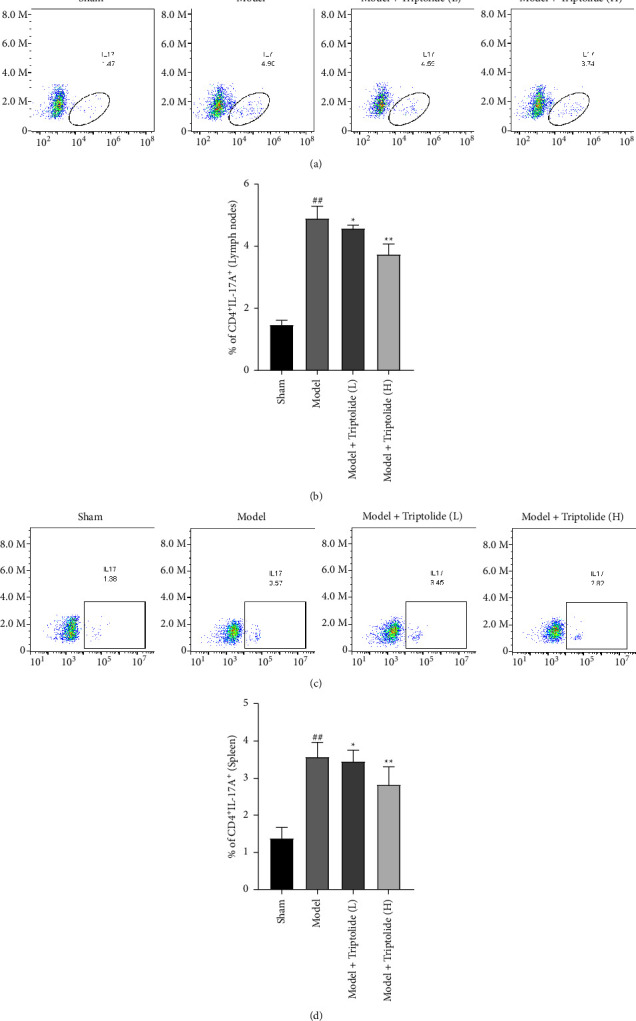
Influences of triptolide on Th17 cells in psoriatic mice induced by IMQ. On the 8^th^ day after triptolide treatment, isolation of draining LN and spleen cells from mouse models in the indicated groups was performed. (a–d) Flow cytometry analysis used to analyze the cells for determining the percentages of IL-17A-expressing cells within the CD4^+^ population. Histograms are gated on the CD4^+^ population. Each column is representative of data obtained from a minimum of 8 animals. ^##^*P* < 0.01 compared to the sham group; ^*∗*^*P* < 0.05, ^*∗∗*^*P* < 0.01 compared to the model group.

Due to the crucial importance of Th17 cell development in the etiology of autoimmune diseases, we wondered about the possibility of triptolide in alleviating psoriasis caused by IMP through repression of Th17 development. Through flow cytometry analysis (Figures 5(a)–5(d)), we observed the increased percentages of CD4^+^IL-17A^+^ in the LNs (4.90 ± 0.42%) and spleens (3.57 ± 0.41%) in the model group relative to the sham group. Importantly, triptolide at low dosage suppressed the percentages of CD4^+^IL-17A^+^ in the LNs and spleens to 4.58 ± 0.13% and 3.45 ± 0.32%, respectively, while a high dosage of triptolide reduced CD4^+^IL-17A^+^ percentages in the LNs and spleens to 3.74 ± 0.36% and 2.82 ± 0.50%, respectively. These data suggested that triptolide treatment inhibits Th17 cell development in psoriasis.

### 3.6. Triptolide Inhibits the Expression of Proinflammatory Cytokines in IMQ-Evoked Psoriasis-Like Mice

**Figure 6:**
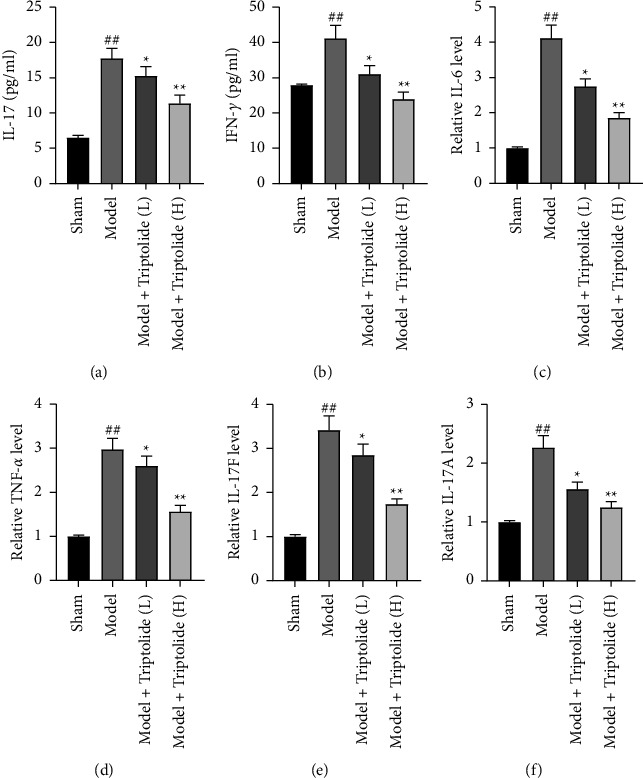
Triptolide suppresses proinflammatory cytokine expression in IMQ-induced psoriatic mice. (a and b) Serum concentrations of IL-17 and IFN-*γ* in IMQ-induced psoriatic mice detected by ELISA after triptolide treatment for 8 days. (c–f) The mRNA expression of proinflammatory cytokines in psoriasis-like lesions also detected via RT-qPCR on day 8. Each column is representative of data obtained from a minimum of 8 animals. ^##^*P* < 0.01 compared to the sham group; ^*∗*^*P* < 0.05, ^*∗∗*^*P* < 0.01 compared to the model group.

To recognize the immunosuppressive influence of triptolide on IMQ-induced skin lesions, the serum concentrations of proinflammatory cytokines (IL-17 and IFN-*γ*) of mouse models in each group were first measured through ELISA. As presented in Figures 6(a) and 6(b), serum IL-17 and IFN-*γ* levels were higher in psoriatic mice induced by IMQ than normal mice in the sham group. After treatment with triptolide, induced upregulation of IL-17 and IFN-*γ* levels in serum of psoriatic-like mice was downregulated by 10 mg/kg triptolide and further reduced by 40 mg/kg triptolide. On the other hand, the expression of several cytokines at mRNA levels was detected by RT-qPCR analysis. Compared with the mice in the sham group, expression levels of Th17-type cytokines, including IL-6, TNF-*α*, IL-17F, and IL-17A, were notably elevated in the skin of IMQ-induced psoriatic mice. However, treatment of psoriatic mice with triptolide resulted in an obvious suppression of expression levels of IL-6, TNF-*α*, IL-17F, and IL-17A in the psoriasis-like lesions in a dose-dependent manner (Figures 6(c)–6(f)). Therefore, triptolide might ameliorate mouse psoriasis evoked by IMQ via inhibiting proinflammatory cytokines.

### 3.7. Triptolide Upregulates miR-204-5p Expression and Inhibits STAT3 Phosphorylation In Vivo

**Figure 7:**
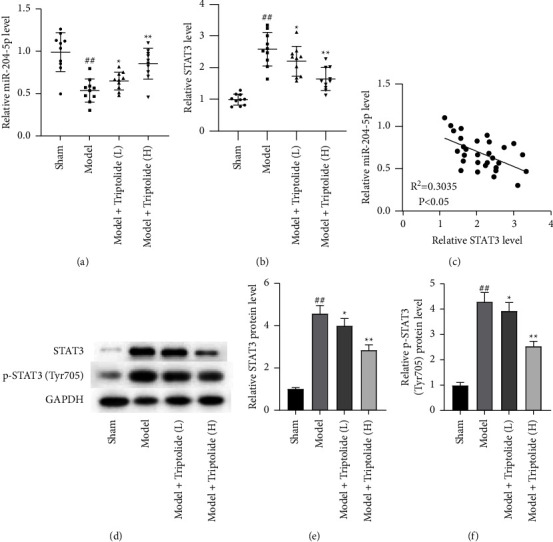
Effects of triptolide on miR-204-5p and STAT3 expression in psoriasis-like lesions. (a and b) After collecting the dorsal skin of mouse models in the indicated groups on the 8th day, RT-qPCR was performed for examining miR-204-5p and STAT3 expression. (c) The expression correlation between miR-204-5p and STAT3 by Spearman's correlation analysis. (d–f) The protein levels of STAT3 and p-STAT3 in the dorsal skin of mouse models tested via Western blot analysis. Each column is representative of data obtained from a minimum of 8 animals. ^##^*P* < 0.01 compared to the sham group; ^*∗*^*P* < 0.05, ^*∗∗*^*P* < 0.01 compared to the model group.

To investigate the role of miR-204-5p and STAT3 in triptolide-mediated psoriasis *in vivo*, we examined the expression of miR-204-5p and STAT3 in healthy skin tissues and triptolide-treated psoriatic lesion skin tissues of mouse models. The results from RT-qPCR analysis demonstrated that miR-204-5p expression was decreased in IMP-induced psoriatic lesion skin tissues relative to healthy skin tissues. Following triptolide treatment, miR-204-5p expression was dose-dependently increased (Figure 7(a)). On the contrary, we found an increase in STAT3 expression in the model group relative to the sham group. Induced STAT3 expression was dose-dependently suppressed by triptolide treatment (Figure 7(b)). Moreover, a negative expression correlation between miR-204-5p and STAT3 in IMP and triptolide-treated psoriatic mice was found (Figure 7(c)). Furthermore, the protein and phosphorylation levels of STAT3 were also detected in psoriatic lesion skin tissues by RT-qPCR, which manifested that elevation of STAT3 protein and phosphorylation levels was reduced by increasing doses of triptolide (Figures 7(d)–7(f)). Conclusively, triptolide elevates miR-204-5p expression and suppresses STAT3 phosphorylation *in vivo*.

## 4. Discussion

Psoriasis is a multifactorial inflammatory skin disorder seriously affecting the physical and psychological health of numerous patients [29]. Recently, an immunosuppressive agent such as cyclosporine or methotrexate has been applied in psoriasis treatment, but it causes severe adverse effects to the liver and kidneys. Compared to conventional drugs, biological anti-inflammatory antibodies which suppress the IL-23/IL-17 axis are much more expensive. Therefore, seeking a more efficient and less expensive drug for psoriasis treatment is important.

As a leading role in psoriasis pathogenesis, Th17 cells have been proven to participate in the formation of IMQ-generated psoriatic lesions in mouse models [30]. Therefore, in this investigation, we focused on detecting the well-known anti-inflammatory effects of triptolide in a mouse model with the aim of further elucidating the mechanism by which triptolide exerts anti-inflammatory influences and protects against the formation of psoriasis-like lesions induced by IMQ. Our findings illuminated that triptolide protected against topical IMQ treatment-induced psoriasis-like lesions in mouse models. Moreover, our study suggested that this protection was achieved by suppressing Th17 cells and triptolide inhibited Th17 response through miR-204-5p-mediated suppression of STAT3 phosphorylation.

Research has validated that the dosage of triptolide under 200 *μ*g/kg shows no discernible adverse effects, while 300–1000 *μ*g/kg of triptolide results in liver injury [31–36], which indicates that triptolide exhibited toxicological or pharmacological effects largely depend on its dosage. In our study, the concentration of triptolide at 1–500 nM was used to treat the isolated splenocytes which showed that 10–50 nM triptolide significantly inhibited cell viability, while triptolide at the dosage of 500 nM led to cytotoxicity. Additionally, the dosage of triptolide under 10 nM suppressed Th17 cell differentiation from splenocytes. Therefore, triptolide might become a promising drug for psoriasis treatment until the identification of the appropriate dosage for patients.

Both human psoriatic plaques and IMQ-induced mouse psoriatic plaques have numerous erythema and scales in terms of histopathological features. Our study demonstrated that following continuous IMQ treatment, significant thickening, erythema, and scaling on the dorsal skin of mouse models at day 8 was seen. Of note, these symptoms were alleviated by triptolide treatment, which is consistent with the results of the previous report [25]. Immune cell infiltration is another key factor in psoriasis development except for epidermal cell hyperplasia. It has been illuminated that the IL-23/IL-17 axis can mediate psoriasis-like skin inflammation caused by IMQ induction in mouse models [37]. Cytokines such as TNF-*α*, IL-22, IL-17A, IL-17F, and IL-6 are produced by IL-23-activating Th17 cells, which promotes the proliferative ability of keratinocytes [38,39]. In the current investigation, compared with sham mice, significantly upregulated levels of Th17 cytokines including IL-6, TNF-a, IL-17F, and IL-17A were identified in skin samples of IMQ-treated mice. Additionally, a greater number of CD4^+^IL-17A^+^ cells was identified in the LNs and spleens of IMQ-induced mouse models while triptolide elicited inhibitive effects on Th17 response, which is in accordance with previous literature studies [28, 40].

STAT3, which is initially identified as acute phase response factor (APRF), is activated in hepatocytes treated by IL-6 [41]. The activation of STAT3 is associated with pathologies such as autoinflammatory or autoimmune conditions including psoriasis [42]. Not surprisingly, STAT3 has been demonstrated as a crucial player in Th17 cell biology, and the differentiation of Th17 cells can be suppressed by STAT3 depletion both *in vivo* and *in vitro* [43,44]. Many documents have proved that triptolide represses STAT3 signaling in multiple human diseases. For example, triptolide contributes to autophagy through reactive oxygen species generation in ovarian cancer SKOV3/DDP cell lines resistant to cisplatin by inhibiting STAT3 signaling [45]. Another report reveals that via suppression of the Janus-activated kinase 2/STAT3 pathway, triptolide suppresses the expression of inflammatory cytokines and inhibits cell proliferative abilities in fibroblast-like synoviocytes stimulated by IL-6/sIL-6R [46]. Consistently, our study manifested that the expression and phosphorylation levels of STAT3 were suppressed by triptolide both *in vivo* and *in vitro*, which indicated that psoriasis development is inhibited by triptolide through suppression of STAT3.

To further prove how triptolide regulates STAT3, we focused on miRNAs which were associated with psoriasis or triptolide. MicroRNAs are short noncoding RNAs that could posttranscriptionally modulate protein-coding gene expression, and emerging miRNAs have been uncovered to be effective in the function of immune cells and keratinocytes in patients with psoriasis [47–49]. MiR-204-5p is a newly identified microRNA, which has been elucidated to inhibit synovial fibroblast inflammation in osteoarthritis [50], and is correlated with CD4^+^ central memory and effector memory T cell infiltration [51]. In addition, the promotive effect of triptolide on miR-204 expression has been verified in S2-VP10 cells [27]. Consistently, our study manifested that miR-204-5p expression was also elevated by triptolide in HaCaT cells and psoriatic lesion skin tissues in a dose-dependent manner. Interestingly, through bioinformatic analysis, the binding sequences between miR-204-5p and STAT3 were predicated, and the combination was proved by luciferase reporter assay. Moreover, it was found that STAT3 expression levels were reduced upon miR-204-5p overexpression and the negative expression correlation between miR-204-5p and STAT3 was recognized. All these findings suggested that triptolide inhibited the phosphorylation of STAT3 via elevating miR-204-5p and thus repressing Th17 response and psoriasis development.

Conclusively, our results manifested that triptolide inhibited Th17 response by upregulating miR-204-5p and suppressing STAT3 phosphorylation in psoriasis. However, the sample size was relatively small, more samples needed to be collected, and further studies were needed to further confirm our findings in the future. Considering the toxicity of triptolide to mouse models, its precise safe dosage to human needs further investigation. Finally, due to the complexity of the molecular mechanism, some upstream molecules or other downstream signaling pathways of the triptolide/miR-204-5p/STAT3 axis deserve further exploration.

## Data Availability

The datasets used or analyzed during the current study are available from the corresponding author upon request.
